# Genetic and Dietary Iron Overload Differentially Affect the Course of *Salmonella* Typhimurium Infection

**DOI:** 10.3389/fcimb.2017.00110

**Published:** 2017-04-11

**Authors:** Manfred Nairz, Andrea Schroll, David Haschka, Stefanie Dichtl, Piotr Tymoszuk, Egon Demetz, Patrizia Moser, Hubertus Haas, Ferric C. Fang, Igor Theurl, Günter Weiss

**Affiliations:** ^1^Department of Internal Medicine II, Infectious Diseases, Immunology, Rheumatology, Pneumology, Medical University of InnsbruckInnsbruck, Austria; ^2^Department of Pathology, Medical University of InnsbruckInnsbruck, Austria; ^3^Division of Molecular Microbiology, Biocenter, Medical University of InnsbruckInnsbruck, Austria; ^4^Department of Laboratory Medicine, University of WashingtonSeattle, WA, USA; ^5^Department of Microbiology, University of WashingtonSeattle, WA, USA

**Keywords:** iron, macrophage, hepcidin, lipocalin, *Salmonella*, infection, siderophore

## Abstract

Genetic and dietary forms of iron overload have distinctive clinical and pathophysiological features. HFE-associated hereditary hemochromatosis is characterized by overwhelming intestinal iron absorption, parenchymal iron deposition, and macrophage iron depletion. In contrast, excessive dietary iron intake results in iron deposition in macrophages. However, the functional consequences of genetic and dietary iron overload for the control of microbes are incompletely understood. Using *Hfe*^+/+^ and *Hfe*^−/−^ mice in combination with oral iron overload in a model of *Salmonella enterica* serovar Typhimurium infection, we found animals of either genotype to induce hepcidin antimicrobial peptide expression and hypoferremia following systemic infection in an Hfe-independent manner. As predicted, *Hfe*^−/−^ mice, a model of hereditary hemochromatosis, displayed reduced spleen iron content, which translated into improved control of *Salmonella* replication. *Salmonella* adapted to the iron-poor microenvironment in the spleens of *Hfe*^−/−^ mice by inducing the expression of its siderophore iron-uptake machinery. Dietary iron loading resulted in higher bacterial numbers in both WT and *Hfe*^−/−^ mice, although Hfe deficiency still resulted in better pathogen control and improved survival. This suggests that Hfe deficiency may exert protective effects in addition to the control of iron availability for intracellular bacteria. Our data show that a dynamic adaptation of iron metabolism in both immune cells and microbes shapes the host-pathogen interaction in the setting of systemic *Salmonella* infection. Moreover, Hfe-associated iron overload and dietary iron excess result in different outcomes in infection, indicating that tissue and cellular iron distribution determines the susceptibility to infection with specific pathogens.

## Introduction

*HFE* encodes an atypical MHC class I molecule which plays a major role in the regulation of iron homeostasis under basal conditions (Feder et al., 1996; Ludwiczek et al., 2004). *HFE* mutations, especially the homozygous *C282Y* substitution, result in type I (AKA classical) hereditary hemochromatosis (HH) (Camaschella et al., 2002; Pietrangelo, 2004; Weiss, 2010), the most frequent form of HH mainly found in people of Northern or Western European ancestry. HH is characterized by reduced serum levels of the antimicrobial peptide Hamp (hepcidin) and increased duodenal absorption of iron via divalent metal transporter 1 (Dmt1) and ferroportin 1 (Fpn1) despite progressive iron overload in parenchymal organs including the liver, pancreas, and heart (Zoller et al., 1999, 2001; Bridle et al., 2003; Pietrangelo, 2004; Bardou-Jacquet et al., 2013). The precise role of the HFE protein, however, remains incompletely understood. HFE binds to transferrin receptor 1 (TfR1) thus lowering its affinity for iron-laden transferrin (Feder et al., 1998; Lebrón et al., 1998; Bennett et al., 2000). This interaction controls cellular iron acquisition while also modifying the expression of the key iron-regulatory hormone Hamp (Ahmad et al., 2002; Nicolas et al., 2003; Ludwiczek et al., 2005; Vujic Spasic et al., 2008). The latter mechanism involves the sensing of circulating iron levels by TfR1 and TfR2, which reciprocally complex with HFE expressed on hepatocytes (Schmidt et al., 2008; Wallace et al., 2009). Mutations in *HFE* (or *TFR2*) impair this iron-sensing mechanism, resulting in the insufficient generation of Hamp and increased iron absorption (Goswami and Andrews, 2006; D'Alessio et al., 2012). Of note, macrophages lacking HFE display an iron-poor phenotype which has been attributed to enhanced iron export (Cairo et al., 1997; Drakesmith et al., 2002; Wang et al., 2003).

Systemic iron availability, erythropoietic iron demand, hypoxia, hormones, and inflammatory signals are key factors that modulate the production of the iron homeostatic regulator Hamp (Nemeth et al., 2004a; Bozzini et al., 2008; Theurl et al., 2010; Armitage et al., 2011; Kautz et al., 2014; Nairz et al., 2014; Canali et al., 2017). Hamp controls iron homeostasis upon binding Fpn1, which triggers Fpn1 internalization, degradation (Nemeth et al., 2004b) and blockade of iron efflux from duodenal enterocytes and macrophages, which recycle iron from senescent erythrocytes. Inflammation-driven Hamp induction thus causes iron sequestration within the mononuclear phagocyte system (MPS), which limits iron availability for extracellular pathogens (Bridle et al., 2003; Ludwiczek et al., 2003; Ganz, 2005; Theurl et al., 2008a).

In infections with the intracellular bacterium *Salmonella enterica* serovar Typhimurium, macrophages constitute an important habitat for pathogen replication and persistence (Malik-Kale et al., 2011). Because many bacteria are highly dependent on a sufficient supply of iron for their growth and pathogenicity, macrophage iron homeostasis is an important determinant of disease outcome (Nairz et al., 2014). On one hand, macrophage iron overload is associated with the inhibition of IFN-γ-driven antimicrobial immune effector pathways such as nitric oxide synthase 2 (Nos2) expression, resulting in impaired control of intracellular microbes (Weiss et al., 1994; Mencacci et al., 1997; Oexle et al., 2003). On the other hand, severe iron depletion of the host may result in reduced generation of ROS, which also impairs host defenses. In parallel, iron withholding from pathogens constitutes an efficient host defense strategy (Soares and Weiss, 2015). However, macrophages also contribute to host defense by the production of T-cell stimulatory cytokines and antimicrobial peptides (Graziadei et al., 1997). One of the latter, lipocalin 2 (Lcn2; also known as neutrophil gelatinase-associated lipocalin, siderocalin or 24p3), is secreted by neutrophils and macrophages in response to LPS, IL-1ß, IL-17, and IL-22 (Flo et al., 2004; Shen et al., 2006). In its best characterized function, Lcn2 captures iron-laden bacterial siderophores, small molecules that are enzymatically synthesized and actively secreted by many microbes to bind ferric iron with extraordinarily high affinity (Bachman et al., 2009). Lcn2-sensitive siderophores include enterobactin, carboxy-mycobactins, and bacillibactin. Upon neutralization of these siderophores, Lcn2 contributes to innate resistance against a range of pathogenic bacteria including enterobacteriaceae, mycobacteria and *Bacillus anthracis* by limiting their access to iron (Flo et al., 2004; Berger et al., 2006).

*Salmonella* Typhimurium, a facultative intracellular microbe, needs to gain sufficient access to host iron resources as a prerequisite for replication and virulence (Leung and Finlay, 1991; Vazquez-Torres et al., 1999). To acquire the metal from the host and within infected macrophages, *Salmonella* has evolved both siderophore-dependent and -independent strategies. *Salmonella* synthesizes catecholate-type siderophores such as enterochelin and salmochelins to capture and internalize ferric iron via siderophore receptors (Bäumler et al., 1998; Rabsch et al., 2003; Fischbach et al., 2005). Alternatively, *Salmonella* can incorporate non-siderophore-bound ionic iron using the Feo transport system. In addition, the SitABCD system, whose primary function is bacterial manganese import, may contribute through low-affinity uptake of iron (Zaharik et al., 2004). All three pathways of bacterial iron uptake are linked to *Salmonella* virulence (Tsolis et al., 1996; Janakiraman and Slauch, 2000; Boyer et al., 2002; Crouch et al., 2008; Kim et al., 2013).

Given the central importance of iron for the growth and proliferation of intracellular pathogens such as *Salmonella* and the important role of Hfe in the regulation of systemic iron balance, we performed experiments to assess the influence of Hfe and/or dietary iron overload on host iron homeostasis and immunity in response to *S*. Typhimurium infection. This is of specific interest because Hfe results in macrophage iron depletion whereas dietary iron overload leads to iron accumulation within the MPS.

## Materials and methods

### *Salmonella* infection *In vivo*

All animal experiments described were performed in accordance with Austrian legal requirements. Design of the animal experiments was approved by the Austrian Federal Ministry of Science and Research (approvals BMWF-66.011/0074-C/GT/2007 and /0154-II/3b/2010). Mice were maintained at the central animal facilities of the Medical University of Innsbruck and given free access to water and food. *Hfe*^−/−^ mice were generated as described (Bahram et al., 1999; Flo et al., 2004), crossed back on a C57BL/6 background for at least 10 generations and transferred to the SPF unit of the local Animal Facility by means of embryonic transfer. *Hfe*^+/−^ mice were intercrossed and offspring were genotyped using the following primers (obtained from Microsynth): *Hfe* fw: 5′-GAATTAACAGGCCGTTTCTAAAG-3′, *Hfe* rev: 5′-CTTGGAGTAGTGGCTCACACT-3′, *Hfe* neo: 5′-GAGATCAGCAGCCTCTGTTCC-3′.

For *in vivo* infection experiments (Supplementary Figure 1), male mice were used at 20–26 week of age and fed either an iron-enriched diet (C1038 from Altromin) supplemented with 25 g/kg carbonyl iron (Sigma) or a standard diet (180 mg Fe/kg, C1000 from Altromin) 3 week before and during infection. Mice were infected i.p. with 500 CFU *S*. Typhimurium strain ATCC14028 suspended in 200 μl PBS. Animals were monitored 3 times daily for 10 days for signs of illness, and moribund mice were euthanized. Forty-eight and ninety-six hours post-infection, mice were randomly selected for the determination of colony counts. Bacterial load in livers and spleens was determined by plating serial dilutions of organ homogenates on LB agar under sterile conditions. Mice selected for the determination of colony counts were not considered for the recording of survival times.

### Blood counts

Blood samples were drawn under anesthesia by retroorbital puncture and collected in heparinized tubes. An aliquot of heparinized blood was used for complete blood count analysis on a Vet-ABC Animal blood counter (Scil animal care company GmbH).

### Measurement of iron parameters

Serum iron was measured using the QuantiChrom Iron Assay kit (BioAssay Systems) according to the manufacturer's instructions. Serum FT was measured by a specific ELISA kit (LifeSpan BioSciences) according to the manufacturer's protocol (Theurl et al., 2016). Total tissue iron content was measured as described (Sonnweber et al., 2012).

### Histology

Histological examinations of tissues were performed on formalin-fixed tissue sections stained with hematoxylin and eosin (HE) according to a standard protocol (Nairz et al., 2011). Images with HE staining were acquired using a Nikon-Eclipse 80i microscope equipped with a 4x objective with a 0.10 numerical aperture. Image acquisition was performed using NIS-Elements BR3 software.

### Cell culture, *Salmonella* infection *In vitro* and determination of bacterial iron acquisition

Thioglycolate-elicited primary peritoneal macrophages were harvested as described (Schleicher et al., 2005) from C57BL/6 mice of indicated genotypes (detailed below), matched for sex and age, and cultured in RPMI (purchased from Biochrom AG) containing 5% heat-inactivated fetal calf serum (FCS; from PAA), 100 U/mL penicillin, 0.1 mg/mL streptomycin and 10 mM HEPES (all from Sigma). After a 24 h incubation period, macrophages were extensively washed with phosphate-buffered saline (PBS purchased from Invitrogen) and incubated in complete RPMI without antibiotics. Only cell preparations of at least 90–95% purity, as determined by F4/80 surface expression in FACS analysis, were used for subsequent experiments. Macrophages were infected with *S*. Typhimurium ATCC14028 at a multiplicity of infection (MOI) of 10.

Measurement of bacterial iron acquisition was performed as described elsewhere (Nairz et al., 2008). Briefly, *Salmonella*-infected macrophages were washed three times and resuspended in serum-free HEPES-buffered RPMI. After the addition of 5 μM ^59^Fe as citrate (NTBI) or loaded onto human apo-transferrin (TBI; Sigma), cells were incubated for an additional 8 h. Intracellular bacilli were harvested according to a modified protocol as described (Olakanmi et al., 2002; Nairz et al., 2008). An aliquot of the bacterial suspension was plated in serial dilutions onto agar plates to quantify released bacteria, while the remaining volume was filtered through centrifugal filter devices with a PDVF membrane of 0.22 μm pore size (Millipore). Filters containing the trapped bacteria were used to measure *Salmonella*-associated ^59^Fe with a γ-counter. No association of ^59^Fe to *S*. Typhimurium that had been heat-inactivated at 70°C for 20 min could be detected.

### RNA extraction and quantitative real-time PCR

Preparation of total RNA and quantification of mRNA expression by Taqman® or SYBR Green® RT-PCR following reverse transcription was performed exactly as described (Crawford et al., 2016). Murine primers and probes (Microsynth), the latter carrying 5′-FAM and 3′-BHQ1 labels, were used as described elsewhere (Ludwiczek et al., 2005; Theurl et al., 2008b). Bacterial primers and probes have been described (Bearson et al., 2008; Crawford et al., 2016).

### Statistical analysis

Statistical analysis was carried out using a SPSS statistical package. Calculations for statistical differences between various groups were carried out by ANOVA and Tukey's correction for multiple tests. Otherwise, a two-tailed unpaired Student's *t*-test was used. For comparison of survival between subgroups, the Wilcoxon (Gehan) statistic was used. Non-parametric variables (CFU and serum FT) were log-transformed prior to testing. *P* < 0.05 was used to determine statistical significance, 0.05 ≤ *P* < 1.0 was considered a statistical trend and depicted.

## Results

### Influence of Hfe, dietary iron challenge and *Salmonella* infection on iron parameters

To better understand the influence of Hfe and dietary iron loading on iron homeostasis and the outcome of infection, we used a well-established model of systemic *Salmonella* infection. Wildtype (WT) C57BL/6 (*Hfe*^+/+^) and congenic *Hfe*^−*/*−^ mice were fed either a standard rodent diet with adequate iron content (IA) or an iron-enriched (IE) diet for 3 weeks prior to and during infection. Congenic WT and *Hfe*^−/−^ mice were then systemically infected with 500 colony-forming units (CFU) of *Salmonella enterica* serovar Typhimurium ATCC14028 (*S*. Typhimurium; *S*. Tm.) via intraperitoneal (i.p.) injection (as delineated in Supplementary Figure 1). Mock-infected controls received a single i.p. injection of PBS as a control (Ctrl.). Animals were monitored for up to 10 days. On days 2 and 4 post-infection, randomly selected animals were sacrificed and bacterial loads (days 2 and 4), erythroid (Supplementary Figures 2B,C) and iron indices as well as the expression of iron metabolic genes (day 2) were evaluated.

**Figure 1:**
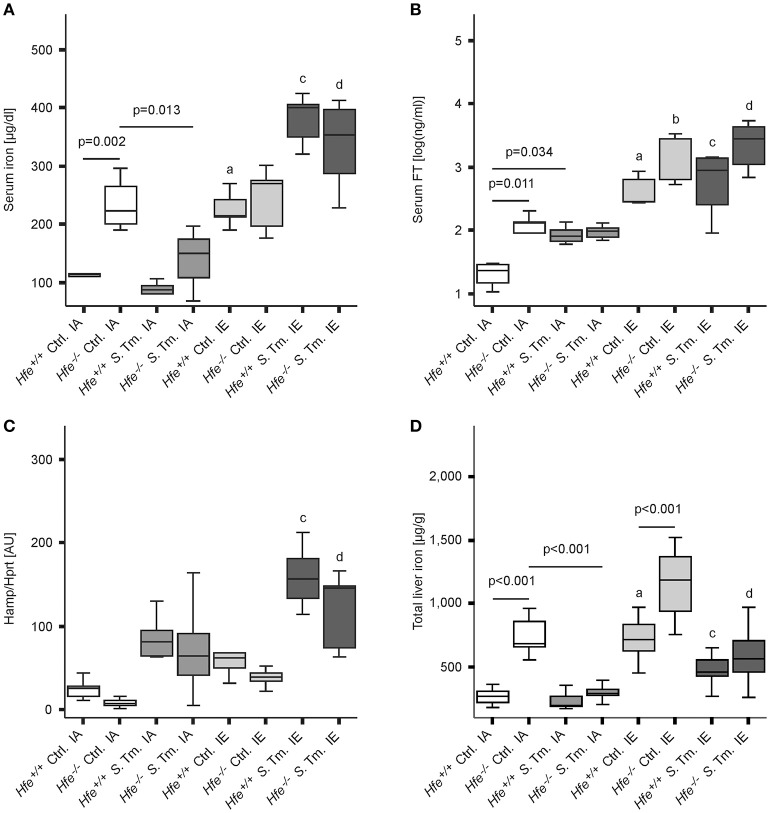
**Influence of Hfe, dietary iron challenge and ***Salmonella*** infection on systemic iron parameters**. *Hfe*^−/−^ and congenic C57BL/6 WT animals (*Hfe*^+/+^) were fed either a standard iron-adequate diet (IA) or an iron-enriched diet (IE) and infected i.p. with 500 CFU of *S*. Typhimurium (*S*. Tm.). Mock-infected controls (Ctrl.) received diluent. Serum iron **(A)** and ferritin (FT) levels **(B)** were measured after 48 h. In parallel, the expression of Hamp mRNA **(C)** in the liver was determined relative to the house-keeping gene Hprt by quantitative RT-PCR. Total liver iron content 48 post-infection was measured colorimetrically and normalized for wet tissue weight **(D)**. Data were compared by means of ANOVA with Tukey's *post hoc* test. Values are depicted as lower quartile, median and upper quartile (boxes), and minimum/maximum ranges. Statistical significant differences within each diet group are indicated. Additional letters represent statistically significant differences (*P* < 0.05) as follows: (a) *Hfe*^+/+^ Ctrl. IA vs. *Hfe*^+/+^ Ctrl. IE; (b) *Hfe*^−/−^ Ctrl. IA vs. *Hfe*^−/−^ Ctrl. IE; (c) *Hfe*^+/+^ *S*. Tm. IA vs. *Hfe*^+/+^ *S*. Tm. IE; *Hfe*^−/−^ *S*. Tm. IA vs. *Hfe*^−/−^ *S*. Tm. IE. *n* = 7–10 per group.

As predicted, under control conditions *Hfe*^−/−^ mice fed an iron-adequate (IA) diet showed elevated serum iron and serum ferritin (FT) levels as compared to *Hfe*^+/+^ controls. Both *Hfe*^+/+^ and *Hfe*^−/−^ responded to systemic infection with a reduction of serum iron concentrations (hypoferremia) (Figure 1A). Of interest, serum iron levels increased upon dietary iron challenge independent of the *Hfe* genotype, as *Hfe*^−/−^ and congenic *Hfe*^+/+^ mice displayed comparable serum iron levels on an IE diet. Intriguingly, *Hfe*^+/+^ and *Hfe*^−/−^ mice maintained on an IE diet prior to and during *Salmonella* infection had even higher serum iron levels as compared to uninfected animals on an IE diet and did not mount a hypoferremic response. Serum FT levels were dramatically increased during dietary iron overload, whereas the stimulatory effect of *Salmonella* infection on serum FT levels was minimal (Figure 1B).

While *Hfe*^−/−^ mice tended to have lower hepatic Hamp mRNA expression as compared to congenic WT mice, the induced Hamp expression in response to infection or dietary iron overload remained intact in *Hfe*^−/−^ mice compared to WT littermates (Figure 1C). As expected, *Hfe*^−/−^ mice had an elevated total iron content in the liver, and the IE diet resulted in hepatic iron accumulation (Figure 1D). Serum IL-6 concentrations were unaffected by the Hfe genotype (Supplementary Figure 2A).

**Figure 2:**
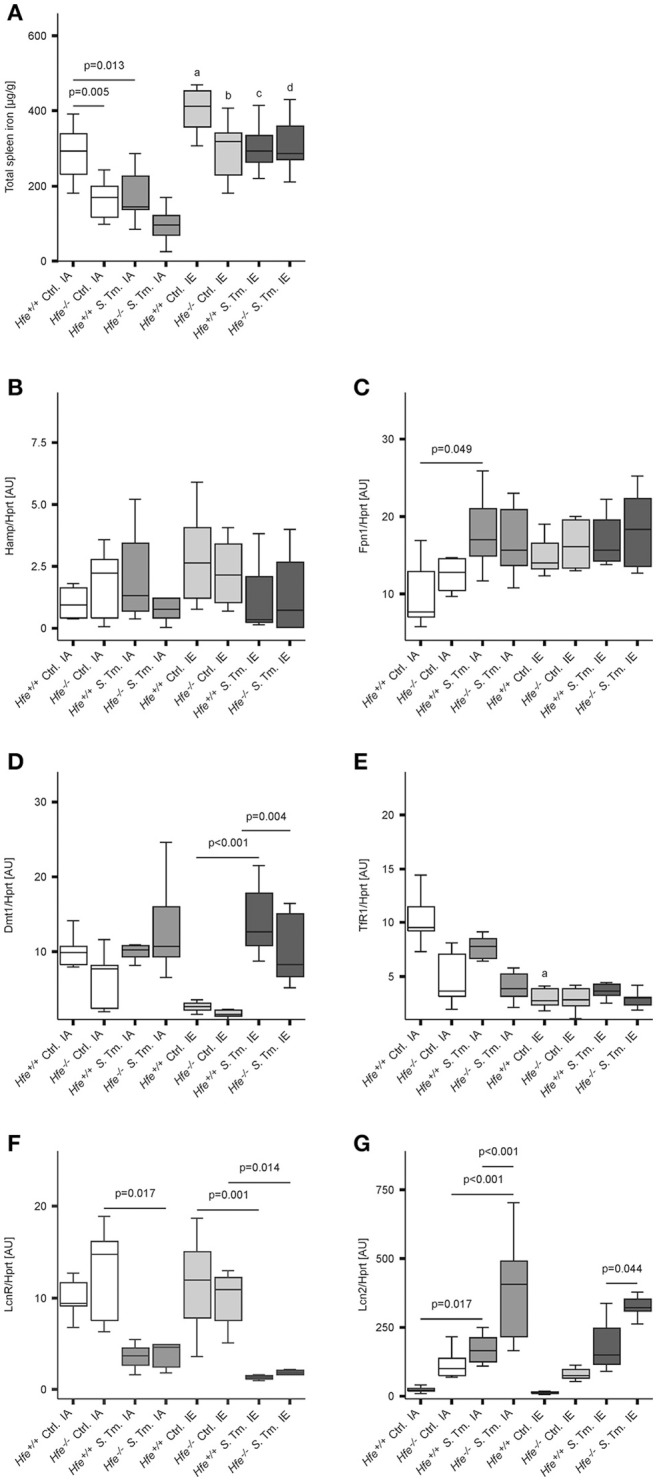
**Influence of Hfe, dietary iron challenge and ***Salmonella*** infection on splenic iron parameters**. Total spleen iron content **(A)** and mRNA levels of iron metabolic genes **(B–G)** in the spleen as determined by quantitative RT-PCR were measured after 48 h. Hamp **(B)**, Fpn1 **(C)**, Dmt1 **(D)**, TfR1 **(E)**, LcnR **(F)**, and Lcn2 **(G)** mRNA levels were determined relative to the house-keeping gene Hprt at baseline and 48 h post-infection. Data were compared by means of ANOVA with Tukey's *post hoc* test. Values are depicted as lower quartile, median and upper quartile (boxes), and minimum/maximum ranges and only statistically significant differences are indicated exactly as described for Figure 1. Additional letters represent statistically significant differences (*P* < 0.05) as follows: (a) *Hfe*^+/+^ Ctrl. IA vs. *Hfe*^+/+^ Ctrl. IE; (b) *Hfe*^−/−^ Ctrl. IA vs. *Hfe*^−/−^ Ctrl. IE; (c) *Hfe*^+/+^ *S*. Tm. IA vs. *Hfe*^+/+^ *S*. Tm. IE; *Hfe*^−/−^ *S*. Tm. IA vs. *Hfe*^−/−^ *S*. Tm. IE. *n* = 7–10 per group.

As previously shown (Cairo et al., 1997; Nairz et al., 2009), *Hfe*^−/−^ mice had reduced total iron content in the spleen is comparison to *Hfe*^+/+^ mice (Figure 2A). Whereas, dietary iron challenge resulted in an increase in spleen iron levels, *Salmonella* infection caused a small yet significant reduction. Splenic Hamp mRNA expression was not significantly affected by either dietary iron content or infection (Figure 2B). In contrast, Fpn1 mRNA expression increased in response to *Salmonella* infection but was not affected by dietary iron overload or Hfe genotype (Figure 2C). Dmt1 and TfR1 mRNA levels were negatively affected by oral iron challenge (Figures 2D,E). Concurrent *Salmonella* infection reverted Dmt1 expression to basal levels, while TfR1 expression remained suppressed. However, there was no substantial influence of *Hfe* genotype on expression of these iron acquisition molecules. Splenic Lcn2 receptor (LcnR) expression was significantly reduced following *Salmonella* infection (Figure 2F), and Lcn2 mRNA expression in the spleen was higher in *Hfe*^−/−^ as compared to congenic WT mice (Figure 2G).

### Influence of dietary iron content on the course of *Salmonella* typhimurium infection in WT and *Hfe*^−/−^ mice

**Figure 3:**
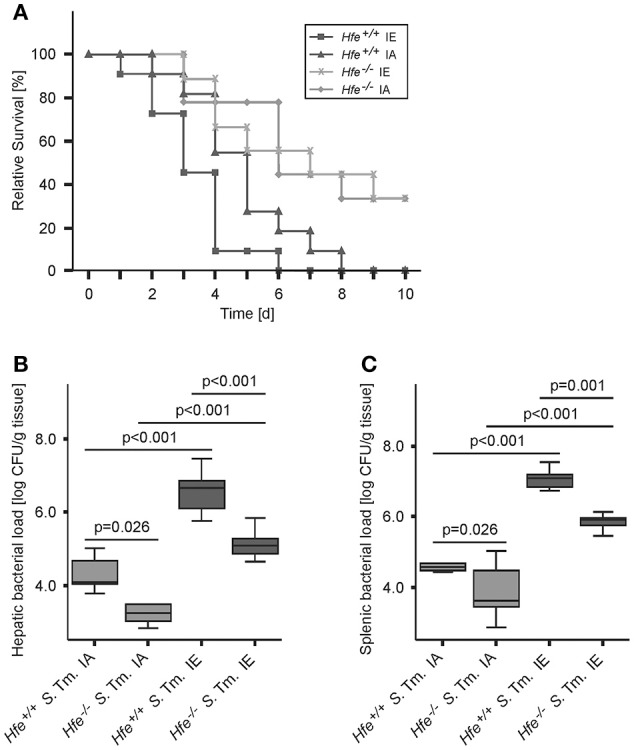
**Influence of dietary iron content on the course of ***Salmonella*** Typhimurium infection in ***Hfe*** WT and ***Hfe***^**−/−**^ mice**. Survival during systemic infection with *Salmonella* Typhimurium was monitored over an observation period of 10 days **(A)**. Data are depicted as Kaplan-Meier curves and were compared by log rank test. *n* = 9–11 per group. Statistically signficant differences are as follows: *P* = 0.031 for *Hfe*^+/+^ IA vs. *Hfe*^−/−^ IA. *P* = 0.028 for *Hfe*^+/+^ IA vs. *Hfe*^+/+^ IE. *P* = 0.954 for *Hfe*^−/−^ IA vs. *Hfe*^−/−^ IE. *P* = 0.002 for *Hfe*^+/+^ IE vs. *Hfe*^−/−^ IE. *P* = 0.041 for *Hfe*^+/+^ IA vs. *Hfe*^−/−^ IE. *P* = 0.003 for *Hfe*^+/+^ IE vs. *Hfe*^−/−^ IA. Bacterial loads of at least 6 animals per group were determined in livers **(B)** and spleens **(C)** of randomly selected animals on d 2 post-infection. CFU data were log-transformed and compared by means of ANOVA with Tukey's *post hoc* test. All statistically significant differences are indicated as lines. Values are depicted as lower quartile, median, and upper quartile (boxes), and minimum/maximum ranges and statistical significances are indicated.

**Figure 4:**
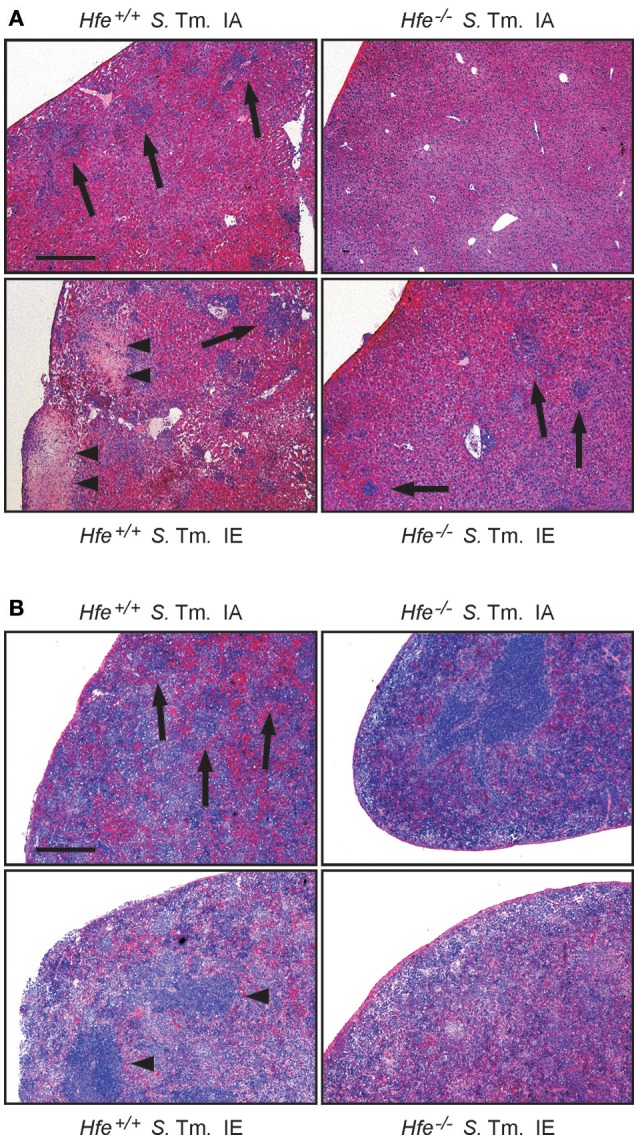
**Influence of dietary iron content on tissue damage during ***Salmonella*** Typhimurium infection in ***Hfe*** WT and ***Hfe***^**−/−**^ mice**. HE-stained sections of livers **(A)** and spleens **(B)** of WT and *Hfe*^−/−^ mice on day 4 of infection show macro-abscesses (arrow heads) in both organs of WT mice on an iron-excessive diet (IE) and scarce inflammatory foci (arrows) in *Hfe*^−/−^ mice fed an iron-adequate diet (IA) with intermediate pathology in the other two treatment/genotype groups. Scale bars: 400 μm.

We next studied the influence of dietary iron overload on disease progression in systemic *Salmonella* infection in *Hfe*^−/−^ and congenic C57BL/6 WT animals. All WT mice died by day 8 of infection independent of their dietary iron content, but animals on an iron-enriched (IE) diet succumbed 1–2 days earlier (Figure 3A). Of note, 33% of *Hfe*^−/−^ mice on an iron-adequate (IA) or iron-enriched (IE) diet (3 of 9 mice in each group) survived the infection beyond day 10 of the observation period. Hepatic and splenic microbial loads of randomly selected animals were quantified on days 2 and 4 of infection. Dietary iron overload significantly increased the bacterial load in both organs in WT mice as well as in *Hfe*^−/−^ animals 2 days post-infection (Figures 3B,C). By day 4 of infection, *Hfe*^−/−^ mice fed an IE diet controlled microbial replication as efficiently as their WT littermates maintained on an IA diet (Supplementary Figures 3A,B). Moreover, tissue sections obtained on day 4 post-infection revealed that WT mice on an IA diet had microabscesses in the liver, which were partly confluent. WT mice on an IE diet also exhibited hepatic macroabscesses. In contrast, hardly any microabscesses were observed in the livers of *Hfe*^−/−^ mice on an IA diet, and only solitary lesions were visible in *Hfe*^−/−^ mice on an IE diet (Figure 4A). Similar observations were made in the spleens of *Salmonella*-infected mice on day 4 post-infection. Only *Hfe*^−/−^ mice on an IA diet had a relatively normal spleen size (Figure 4B). These histopathologic findings were accompanied by corresponding alterations in spleen weight (Supplementary Figures 4A,B).

### Classical innate immune functions are Hfe-independent

**Figure 5:**
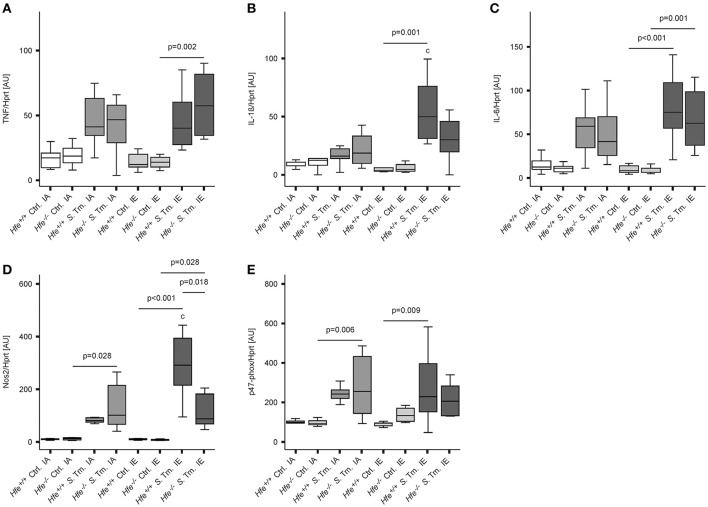
**Classical innate immune functions are Hfe-independent**. The expression of TNF-α **(A)**, IL-1ß **(B)**, IL-6 **(C)**, Nos2 **(D)**, and the p47 phox subunit **(E)** in the spleen was determined relative to the housekeeping gene Hprt by quantitative RT-PCR. Data were analyzed and presented exactly as described in the legend to Figure 1. All statistically significant differences are indicated. Additional letters represent statistically significant differences (*P* <0.05) as follows: (c) *Hfe*^+/+^ *S*. Tm. IA vs. *Hfe*^+/+^ *S*. Tm. IE. *n* = 7–10 per group.

To better define the role of Hfe and dietary iron overload in innate immune function, cytokine and antimicrobial effector system expression was measured on day 2 post-infection. Splenic mRNA levels of TNF, IL-1ß, IL-6, Nos2, and the p47 subunit of the NADPH oxidase (phox) were not affected by the Hfe-genotype (Figures 5A–E). Increased expression of NOS2 and IL-1ß in mice receiving an IE diet relative to those receiving an IA diet paralleled the increased number of bacteria isolated from the spleens of these groups.

### *Salmonella* adapts to the iron-restricted myeloid compartment of *Hfe*^−/−^ mice

**Figure 6:**
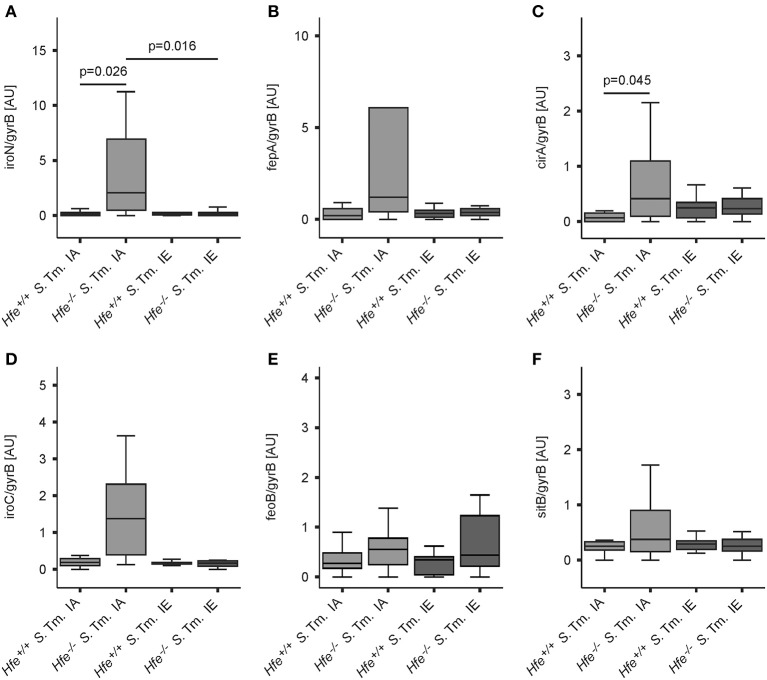
*****Salmonella*** adapts to the iron-restricted myeloid compartment of ***Hfe***^**−/−**^ mice**. The expression of bacterial iron metabolic genes in the spleen was measured by qPCR. Expression of *iroN* **(A)**, *fepA* **(B)**, *cirA* **(C)**, *iroC* **(D)**, *feoB* **(E)**, and *sitB* **(F)** was determined relative to the housekeeping gene *gyrB*. Data were compared by means of ANOVA with Tukey's *post hoc* test. Values are depicted as lower quartile, median and upper quartile (boxes), and minimum/maximum ranges and statistical significances are indicated. *n* = 14–17 per group.

The expression of bacterial iron uptake genes was measured in the spleens of *Salmonella*-infected mice on day 2 post-infection. We found that multiple genes involved in iron uptake were expressed at higher levels in the spleens of *Hfe*^−/−^ mice on an IA diet as compared to the spleens of WT mice receiving the same diet. These genes encoded outer membrane siderophore receptors IroN, FepA, and CirA, as well as the siderophore exporter IroC (Figures 6A–D). In contrast, expression of FeoB (Figure 6E) and SitB (Figure 6F) were not substantially affected by the Hfe status of the host. Notably, no differential induction of bacterial iron genes was observed when WT and *Hfe*^−/−^ mice were on an IE diet, which resulted in splenic iron overload.

**Figure 7:**
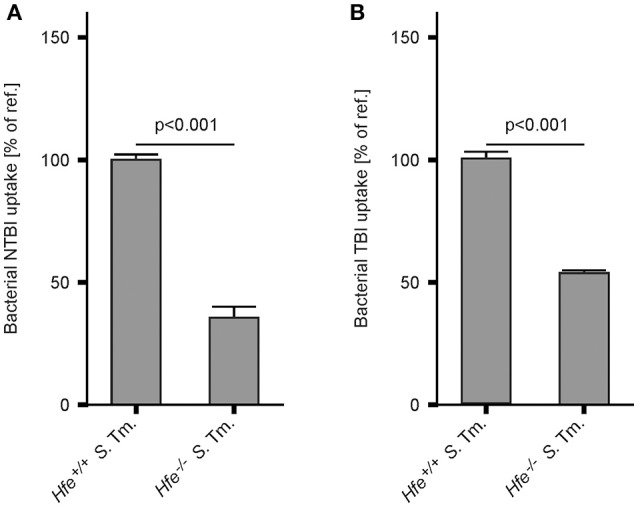
*****Hfe***^**−/−**^ macrophages more efficiently restrict iron from bacteria**. WT and *Hfe*^−/−^ macrophages were infected with *S*. Typhimurium and exposed to ^59^Fe for 24 h. Intracellular bacterial uptake of NTBI **(A)** and TBI **(B)**, respectively, was determined in ^59^Fe-transport studies. The data were compared by a two-tailed unpaired Student's *t*-test and are shown as mean ± S.E.M of at least 3 independent experiments.

In keeping with the induction of siderophore-mediated iron uptake pathways in *Salmonella* residing in the spleens of IA diet-fed *Hfe*^−/−^ mice *in vivo*, we observed that the uptake of host derived ^59^Fe by intracellular *Salmonella*, provided as NTBI or TBI, was reduced in *Hfe*^−/−^ peritoneal macrophages infected *in vitro* relative to congenic WT macrophages (Figures 7A,B).

## Discussion

*Salmonella* Typhimurium causes a systemic disease in mice characterized by a tropism for and replication within professional phagocytes (Richter-Dahlfors et al., 1997; Coburn et al., 2007). *S*. Typhimurium invades its preferred host cell type both by phagocytic uptake and active invasion (Pfeifer et al., 1999). Virulence factors such as those encoded by *Salmonella* Pathogenicity Island-2 are essential for both intracelullar survival and virulence (Hensel et al., 1995, 1998), suggesting that the ability to infect and replicate within macrophages provides a major benefit for the pathogen (Leung and Finlay, 1991). Nutrient availability within this host cell niche is therefore an important factor in *Salmonella* pathogenesis (Carver, 2014).

Iron is one of the essential nutrients that hold a central position in the interplay of host and pathogen (Weinberg, 1974; Schaible and Kaufmann, 2005; Skaar, 2009; Nairz et al., 2010; Drakesmith and Prentice, 2012; Ganz and Nemeth, 2015; Soares and Weiss, 2015). Sufficient access to this trace element is therefore a major determinant of the outcome of *Salmonella* infection. In general, the host response to any bacterial infection involves the restriction of serum iron levels (hypoferremia) through a combined limitation of intestinal iron absorption and macrophage iron recycling. Hamp and its receptor Fpn1 are primary mediators of the hypoferremia, and thereby influence *Salmonella-*host interactions (Nairz et al., 2013; Kim et al., 2014; Armitage et al., 2016). However, a range of additional genetic and environmental factors also influence bacterial iron availability during *Salmonella* infections.

The data presented herein suggest that both the local and systemic availability of iron within the mammalian host affect infection outcome (Nairz et al., 2015a). Hfe deficiency results in reduced Hamp production and increased serum iron levels (Pietrangelo, 2004). Enhanced bacterial replication might be anticipated in an Hfe-deficient host as a result of increased iron availability, and this has been experimentally demonstrated for pathogens such as *Vibrio vulnificus, Yersinia enterocolitica*, and *Yersinia pestis* in the setting of HH (Quenee et al., 2012; Arezes et al., 2015; Miller et al., 2016). However, the opposite is true in the context of infection with an intracellular bacterium such as *S*. Typhimurium because Hfe-deficient macrophages are iron-poor and thus provide an inferior niche for bacterial replication (Nairz et al., 2009). We find that Hfe-deficient macrophages restrict the availability of both non-transferrin-bound (NTBI) and transferrin-bound (TBI) iron to intracellular *Salmonella* more efficiently than WT macrophages. This suggests that the underlying mechanism is independent of Dmt1 and TfR1 and may be attributable to differential iron turnover or efflux. While Fpn1 constitutes the primary pathway for the cellular release of ferrous iron, iron may also be exported via alternative pathways (Devireddy et al., 2005; Keel et al., 2008; Du and Galán, 2009; Nairz et al., 2015b; Lok et al., 2016).

Slc11a1 (also known as Nramp1) has long been known to influence the course of infection with *S*. Typhimurium and certain species of *Mycobacterium* and *Leishmania* (Vidal et al., 1993; Atkinson et al., 1997; Blackwell et al., 2003). Although these pathogens are taxonomically unrelated, they share the features of infecting macrophages, persisting in phagolysosomes and depending on iron. Slc11a1 is incorporated into the phagolysosomal membrane and shifts iron and other divalent ions out of this compartment, thus withdrawing it from phagocytosed microbes (Vidal et al., 1995; Jabado et al., 2000; Wyllie et al., 2002; Fritsche et al., 2007; Valdez et al., 2008). We used C57BL/6 mice for our studies, which carry two dysfunctional Slc11a1 alleles. Therefore, the phenotypes observed in our studies cannot be attributed to this transporter. Moreover, our findings are unlikely to be specific for infections with *S*. Typhimurium but are also relevant to other iron-dependent intracellular pathogens such as *Chlamydia, Legionella* and *Listeria* (Paradkar et al., 2008; Bellmann-Weiler et al., 2010, 2013; Haschka et al., 2015), as well as *Mycobacterium* and *Leishmania*. Accordingly, we note that Hfe deficiency impairs the growth of *Mycobacterium tuberculosis* in human macrophages (Olakanmi et al., 2007).

The iron content of macrophages is influenced by several mechanisms including iron levels in the extracellular microenvironment, expression of iron importers, and exporters, and the rates of erythrophagocytosis and heme-iron recycling (Canonne-Hergaux et al., 1999; Mitterstiller et al., 2016; Theurl et al., 2016). Our models of genetic (i.e., Hfe-associated) and dietary iron overload had different effects on macrophage iron content. Long-term oral iron overload results in increased iron content in virtually all cell types expressing Dmt1 and/or TfR1. In contrast, Hfe deficiency spares the myeloid compartment from iron. We found that oral iron overload results in an increased bacterial load in the spleens and livers of *Salmonella*-infected mice and that Hfe deficiency reduces the bacterial load. Of note, oral iron overload in the setting of Hfe deficiency resulted in intermediate pathogen numbers in spleen and liver on days 2 and 4 of infection. In contrast, the survival of *Hfe*^−/−^ mice over a 10 day period was not affected by dietary iron. *Hfe*^−/−^ mice with dietary iron overload remain more resistant to *Salmonella* infection than WT mice receiving the same diet, with reduced organ loads and increased survival (Figure 3) despite the alleviation of bacterial iron-deprivation by dietary iron supplementation as measured by siderophore gene expression (Figure 6). Albeit somewhat unexpected, these findings suggest that in the later stages of *Salmonella* infection, Hfe plays an immunoregulatory function that is independent of its effect on bacterial iron-restriction. However, the expression of innate immune genes known to mediate host defense against *S*. Typhimurium was not different between *Hfe*^−/−^ and *Hfe*^+/+^ mice (Vazquez-Torres et al., 2000; Vázquez-Torres et al., 2001), nor were differences in T cell-mediated pathways associated with immunity against *S*. Typhimurium such as IL-12, IFN-γ, IL-17, or IL-22, observed (data not shown) (Berger et al., 2006; Raffatellu et al., 2008; Saiga et al., 2008; Schulz et al., 2008; Chan et al., 2009; Godinez et al., 2009; Srinivasan et al., 2012). Given that Lcn2 was expressed at higher levels in *Hfe*^−/−^ mice, it is possible that one of the siderophore-independent effects of Lcn2 may play a role. Lcn2 is a chemoattractant for neutrophils, but this is unlikely to account for a survival difference beyond day 3 of infection (Schroll et al., 2012). Furthermore, Lcn2 promotes macrophage antibacterial effector mechanisms including TNF, IL-6, and Nos2, but these were not observed to be differentially expressed on day 2 or 4 of infection of *Hfe*^−/−^ and *Hfe*^+/+^ mice (Nairz et al., 2015b). It is conceivable that the survival of mice in the late stages of systemic *Salmonella* infection is directly or indirectly influenced by the intestinal microbiome, which is modulated by Lcn2 (Raffatellu et al., 2009; Deriu et al., 2013; Moschen et al., 2016). Alternatively, the comparable survival of *Hfe*^−/−^ mice on an IE on IA diet may involve an Lcn2-independent mechanism beyond innate immunity. For instance, Hfe deficiency may have beneficial effects on apoptosis, ferroptosis, autophagy, or the oxidative stress response within or outside of the myeloid compartment that is independent of dietary iron. If a vital organ system were to be involved, an effect on host survival would be a plausible. An unbiased approach such as RNA-sequencing may be required to identify such a mechanism.

A central and novel finding of our study is that both the host and the microbe adapt their iron metabolism during infection. *S*. Typhimurium expressed genes required for siderophore-mediated iron uptake *in vivo* in the iron-poor spleens of *Hfe*^−/−^ mice. This induction was specifically abrogated by dietary iron overload. This observation raises the question whether virulence factors other than siderophore genes may have been repressed in the setting of dietary iron overload to enhance the survival of *Hfe*^−/−^ mice. This possibility is supported by the known cross-regulation of bacterial iron homeostasis and virulence gene expression (Zaharik et al., 2002). Our data on the upregulation of bacterial iron uptake genes are further in line with the specific induction of iron import mechanisms reported for *Neisseria gonorrhoeae* residing within human monocytes (Zughaier et al., 2014). Both studies thus support the concept that both host myeloid cells and facultatively intracellular bacteria actively compete for iron as essential nutrient.

In summary, the present study highlights the central role of macrophage iron homeostasis in the outcome of infections with iron-dependent intracellular microbes and the differential effects of genetic and dietary iron overload. We also demonstrate that Hfe is not required for the induction of hypoferremia in infected animals on an iron-replete diet. Nevertheless, the *Hfe* mutation alters the iron content of macrophages, which renders the host more resistant to infections with the intracellular pathogen *S*. Typhimurium. The selective pressure imposed by intracellular pathogens may have contributed to the evolutionary conservation of the *HFE C282Y* mutation, accounting for its high allelic frequency in Caucasians (Datz et al., 1998; Moalem et al., 2004).

## Author contributions

MN planned and performed experiments, analyzed the data and wrote the manuscript. AS, DH, SD, PT, ED, and PM performed experiments. HH, FF, and IT interpreted results and edited the manuscript. GW conceived the study, analyzed the data and wrote the manuscript.

### Conflict of interest statement

The authors declare that the research was conducted in the absence of any commercial or financial relationships that could be construed as a potential conflict of interest.
