# Supplementary material for: Genetic and Dietary Iron Overload Differentially Affect the Course of Salmonella Typhimurium Infection
Source: Front Cell Infect Microbiol. 2017 Apr 11;7:110. doi: 10.3389/fcimb.2017.00110 (PMC5387078; doi:10.3389/fcimb.2017.00110)
Supplement: Supplementary file 1 [file Presentation1.pdf]

Supplementary figure 1.

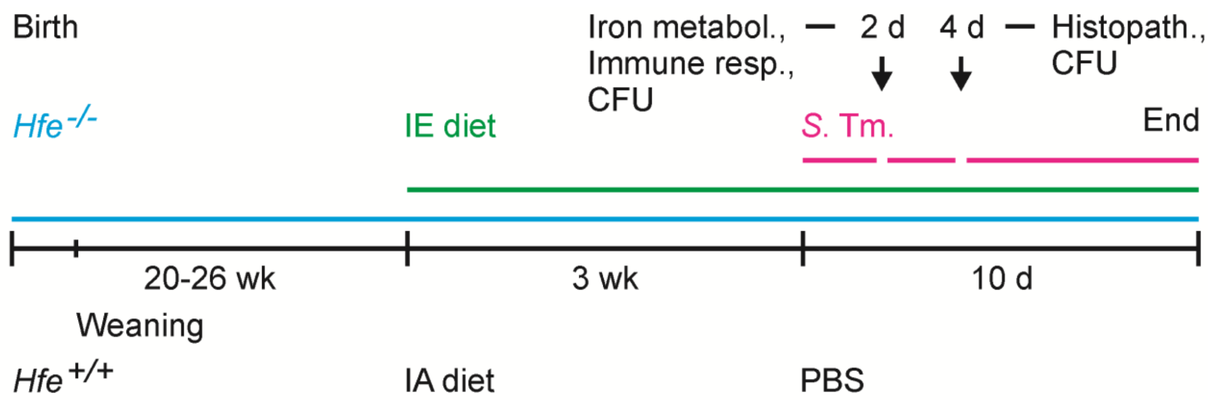

Supplementary figure 2.

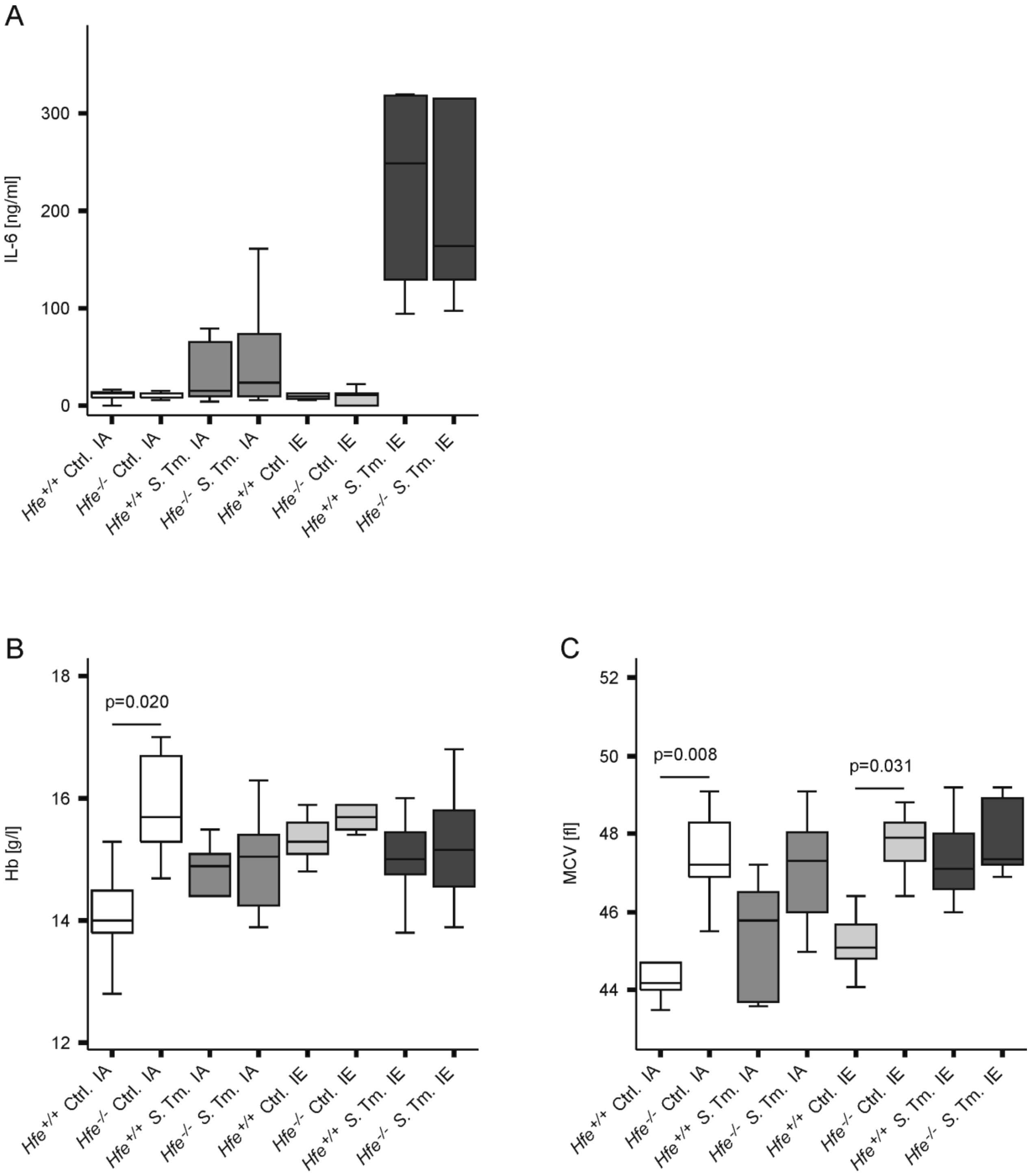

Supplementary figure 3.

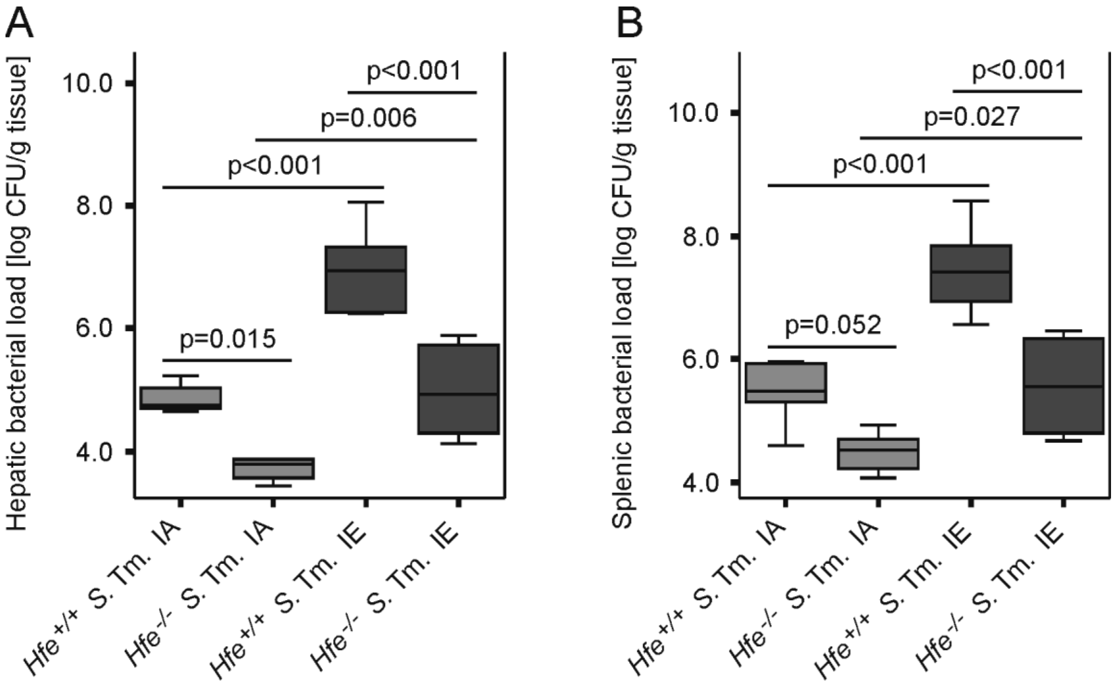

Supplementary figure 4.

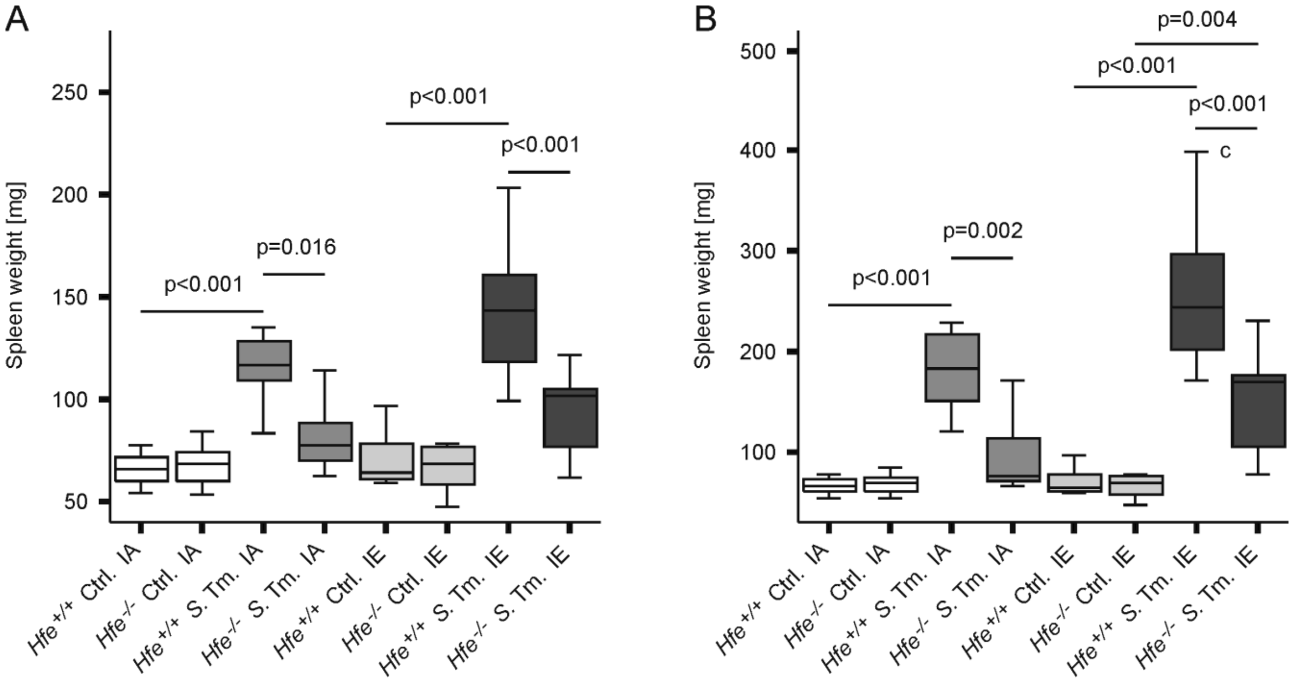

Supplementary figure 1.

Experimental design.

Male *Hfe*$^{-/-}$ and congenic C57BL/6 WT animals (*Hfe*$^{+/+}$) were weaned at 4 wk of age and then kept on a standard iron-adequate (IA) rodent diet before enrollment in the study at 20-26 wk of age. Subsequently, mice were either maintained on the IA diet or switched to an iron-enriched diet (IE) for 3 wk prior to and during the actual infection. Mice were then infected i.p. with 500 CFU of *S.* Typhimurium (*S.* Tm.) or injected with phosphate-buffered sailine (PBS) as diluent. Mice were monitored for up to 10 d. On d 2 (48 h) and d 4 (96 h) after the inoculation, randomly selected mice were sacrificed to enumerate colony-fomring units (CFU) of bacteria (d 2 and d 4) and study iron metabolism and the immune response (d 2) as well as histopathologic findings (d 4). The expression of bacterial iron metabolic genes was also evaluated on d 2 post-infection. Note that the x-axis representing the timeline has an arbitrary rather than linear scale.

Supplementary figure 2.

Serum IL-6, hemoglobin and mean corpuscular volume

Hfe$^{-/-}$ and congenic C57BL/6 WT animals (*Hfe*$^{+/+}$) were fed either a standard iron-adequate diet (IA) or an iron-enriched diet (IE) and infected i.p. with 500 CFU of *S.* Typhimurium (*S.* Tm.). Mock-infected controls (Ctrl.) received diluent. Serum IL-6 levels (A), hemoglobin (Hb) concentration (B), and mean corpuscular volume (MCV), an erythrocyte index (C), were measured at baseline (PBS) and 48 h after infection. Data are presented and compared exactly as in figure 1. Intriguingly, hemoglobin (Hb) levels were unaffected by iron and infection, although under steady-state conditions, *Hfe*$^{-/-}$ mice tended to have higher Hb levels as compared to congenic WT mice (Suppl. fig. 2B). Similarly, erythrocytes of *Hfe*$^{-/-}$ mice had higher mean corpuscular volumes (MCV) as compared to WT mice (Suppl. fig. 2C).

Supplementary figure 3.

Bacterial load in livers and spleens on d 4 of infection

Bacterial loads of at least 6 animals per group were determined in livers (A) and spleens (B) of randomly selected animals on d 4 post-infection. CFU data were log-transformed and compared by means of ANOVA with Tukey's post hoc test. All statistically significant differences are indicated as lines as in figure 3.

Supplementary figure 4.

Spleen weight on d 2 and d 4 of infection as a measure of disease severity

Hfe$^{-/-}$ and congenic C57BL/6 WT animals (*Hfe*$^{+/+}$) were fed either a standard iron-adequate diet (IA) or an iron-enriched diet (IE) and infected i.p. with 500 CFU of *S.* Typhimurium (*S.* Tm.). Mock-infected controls (Ctrl.) received diluent. Spleen weights of at least 6 mice per group were determined on d 2 (A) and d 4 (B) post-infection. Data are presented and compared as in figure 1.
